# Retraction: The effects of sildenafil in maternal and fetal outcomes in pregnancy: A systematic review and meta-analysis

**DOI:** 10.1371/journal.pone.0310291

**Published:** 2024-09-06

**Authors:** The PLOS ONE Editors

After this article [1] was published, two studies included in the meta-analysis were retracted ([2–5]; cited as references 24 and 26 in [1]).

The main conclusion of [1] is that sildenafil usage during pregnancy is associated with increased fetal weight at birth. The authors of [1] redid the meta-analysis with [2] and [3] excluded, and found that the reported conclusion was not supported. This was confirmed by a member of the *PLOS ONE* Editorial Board.

In light of the above concern, the *PLOS ONE* Editors retract this article.

RDdSF, RN, and WMB agreed with the retraction. RS and SP either did not respond directly or could not be reached.
